# Dataset Design for Building Models of Chemical Reactivity

**DOI:** 10.1021/acscentsci.3c01163

**Published:** 2023-12-08

**Authors:** Priyanka Raghavan, Brittany C. Haas, Madeline E. Ruos, Jules Schleinitz, Abigail G. Doyle, Sarah E. Reisman, Matthew S. Sigman, Connor W. Coley

**Affiliations:** † Department of Chemical Engineering, Massachusetts Institute of Technology, Cambridge, Massachusetts 02139, United States; ‡ Department of Chemistry, University of Utah, Salt Lake City, Utah 84112, United States; ¶ Department of Chemistry & Biochemistry, University of California, Los Angeles, Los Angeles, California 90095, United States; § Division of Chemistry and Chemical Engineering, California Institute of Technology, Pasadena, California 91125, United States; ∥ Department of Electrical Engineering and Computer Science, Massachusetts Institute of Technology, Cambridge, Massachusetts 02139, United States

## Abstract

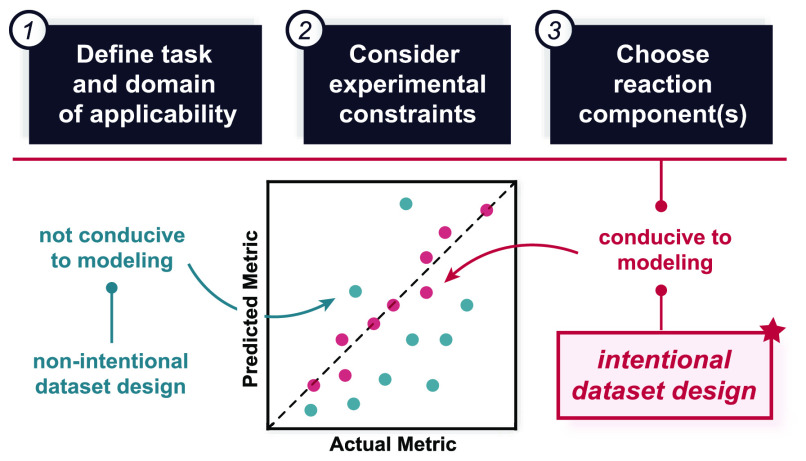

Models can codify our understanding of chemical reactivity and serve a useful purpose in the development of new synthetic processes via, for example, evaluating hypothetical reaction conditions or in silico substrate tolerance. Perhaps the most determining factor is the composition of the training data and whether it is sufficient to train a model that can make accurate predictions over the full domain of interest. Here, we discuss the design of reaction datasets in ways that are conducive to data-driven modeling, emphasizing the idea that training set diversity and model generalizability rely on the choice of molecular or reaction representation. We additionally discuss the experimental constraints associated with generating common types of chemistry datasets and how these considerations should influence dataset design and model building.

## Abstract 2

Dataset design is the construction of experimental datasets with modeling applications in mind. We discuss how modeling considerations should directly influence dataset design, some observed pitfalls, and our recommendations.

## Introduction

Data-driven modeling in organic chemistry dates back almost a century.^1^ Since then, researchers have explored various approaches to correlate molecular properties with reaction performance by using a broad range of techniques from linear free energy relationships (LFERs) to multivariate linear regression to deep learning. Besides the type of model itself, approaches have varied with respect to their application domain, diversity of inputs, and performance measure or prediction target. Here, we focus on models that are trained on experimental data to anticipate quantitative performance metrics, such as reaction yields, selectivities, or even rates.

The major themes and trends in building such structure–property relationships^2,3^ and the broader landscape of predictive chemistry^4^ have been the subject of recent reviews. However, in addition to the many publicized success stories using models to predict the performance of chemical reactions, we have witnessed many cases where modeling has been less successful. Our ability to train models that support chemistry objectives is dependent on data in ways that may be underappreciated and underreported.

**Figure 1:**
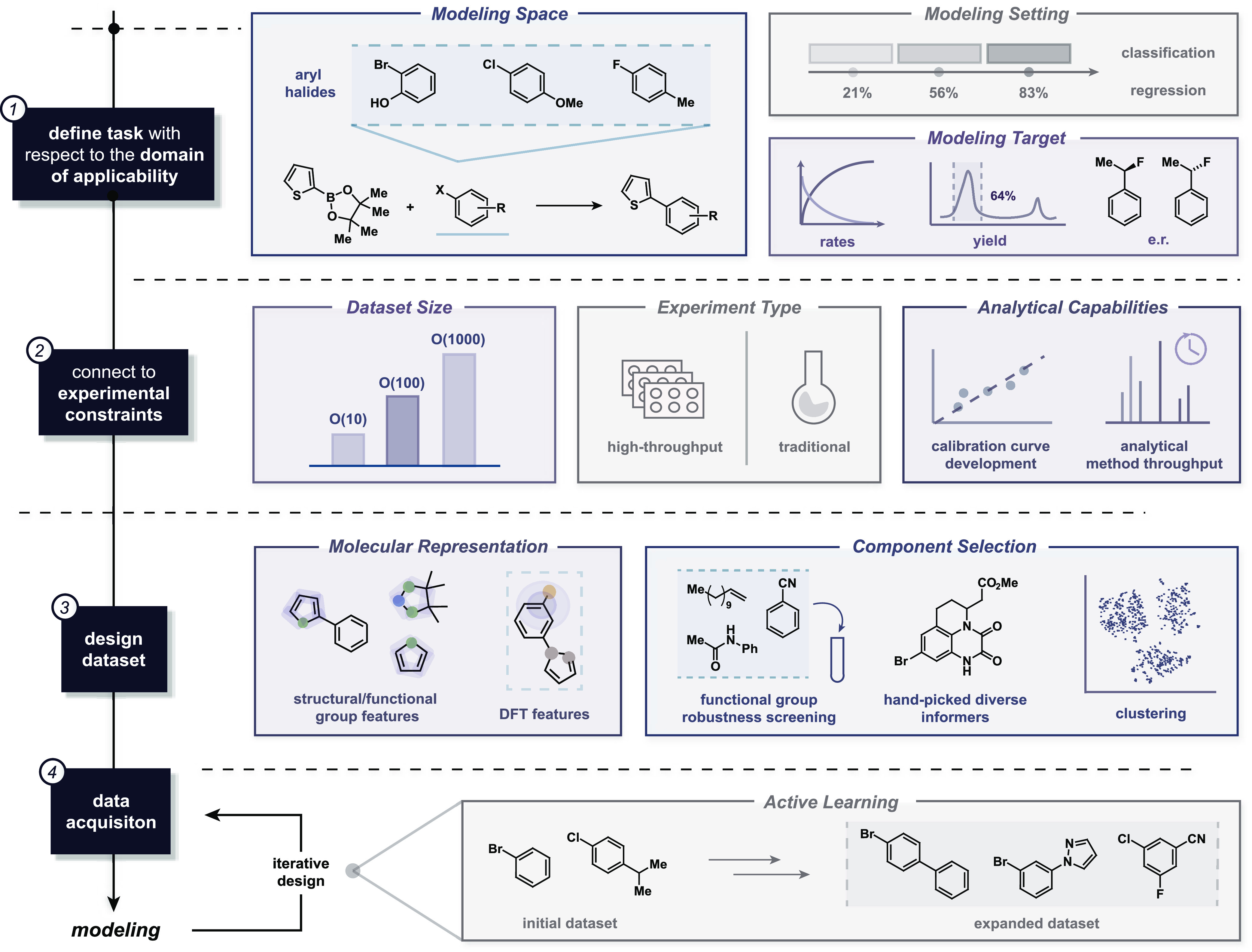
Recommended conceptual workflow for dataset design. From top to bottom, (1) task definition with respect to the modeling space, setting, and target; (2) experimental constraints, including the number of reactions and throughput of the analytical method; and (3) intentional dataset design, emphasizing feature-based reaction component selection.^5,6^ These steps culminate in (4) data acquisition and modeling, with an optional active learning loop for iterative dataset expansion.

In this Outlook, we discuss the concept of dataset design (Figure 1)—the construction of experimental datasets with modeling applications in mind—and some of the pitfalls that we have encountered when learning from datasets that have not been intentionally designed for machine learning. We have organized our discussion around the primary considerations when the aim is model building and describe at each stage how those model considerations should directly influence dataset design.

## Defining the Desired Domain of Applicability

A primary consideration of model building is the desired domain of applicability: the range of inputs over which we would like a model to make accurate predictions. Do we want to be able to query the model with any set of reactants, conditions, and products and have it estimate the yield? Or, are there specific combinations of known substrates that we want to study? Is it acceptable to assume a constant, unvarying temperature and reaction time, or do we also want to understand how those factors influence the reaction performance? Here, we can draw a distinction between “global” and “local” models. The former might involve using a corpus of literature data (for example, the Chemical Abstracts Service (CAS) Content Collection or the Pistachio, USPTO, or Reaxys datasets) containing millions of examples and spanning thousands of reaction types. The latter might involve focusing on a single reaction type and a well-defined set of substrates and reaction conditions; in most substrate scope studies, the reaction conditions are not varied. While a globally useful model is appealing in its scope, it is generally advantageous to have a sufficiently narrow domain of applicability to minimize underlying mechanism changes, reactivity cliffs, or interaction effects in the dataset. These are factors that not only increase modeling difficulty but also are seldom accounted for in model inputs. This perhaps explains why predicting selectivity has seen more consistent success than predicting yield, as is discussed later. Furthermore, some literature-derived datasets are algorithmically extracted from text and have not undergone extensive manual curation or validation, so certain fields may be omitted or incorrect.

**Figure 2:**
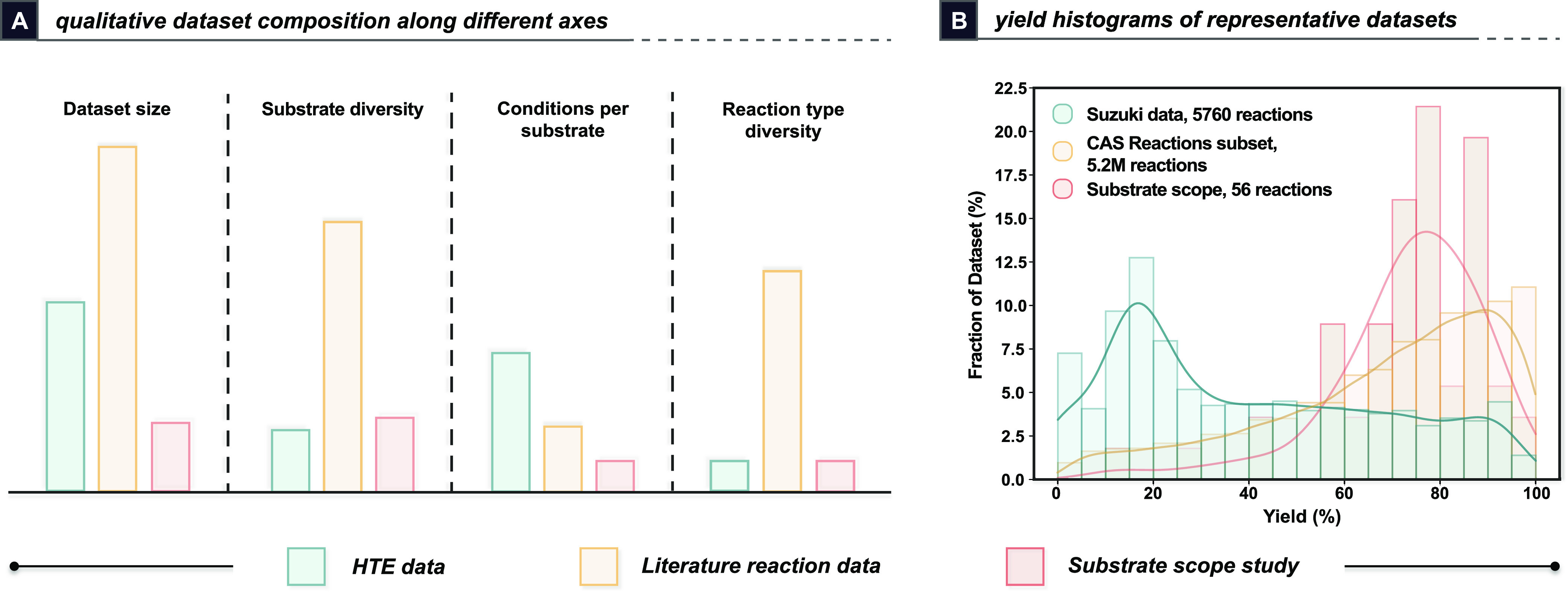
Common types of reaction datasets and their attributes: HTE datasets, literature databases, and substrate scope studies. (A) Each dataset type qualitatively placed within axes of size, substrate diversity, unique conditions per substrate, and reaction type diversity. (B) Yield distribution histograms for a sample dataset of each type: Suzuki HTE data from Pfizer,^8^ a subset of the CAS Content Collection covering published single-step reactions from 2010 to 2015, and a reported reaction scope for the preparation of benzamides.^11^

The datasets we can use for model training exhibit diversity along different axes (Figure 2A). Data derived from the published literature span a wide range of substrates and reaction types, but each reactant–product combination might be reported only once or twice. In contrast, public datasets from high-throughput experimentation (HTE) exist only for a few reaction types so far (Buchwald–Hartwig amination^7^ and Suzuki coupling^8^ being the most popular datasets), although more varied datasets, both in terms of reaction types and design workflow, are emerging.^9,10^ Most HTE datasets are generated through parallel plate-based chemistry in 24-, 96-, 384-, or even higher density well formats. In these experimental campaigns, some reaction variables are easy to vary via automated liquid handling capabilities (e.g., the diversity of concentrations and the combinations of additives), while other aspects (e.g., heterogeneous reactants and the diversity of solvents) are harder to vary given the practical challenges of stock solution preparation.

Acquiring and screening a large number of diverse substrates is the most salient challenge that tends to limit the number of distinct components used in HTE campaigns, which often leverage the combinatorial nature of discrete variable selection. For example, the C–N coupling dataset from Ahneman et al.^7^ covers 4140 reactions defined by the combination of 15 choices for the aryl halide, 23 additives, 4 Pd catalysts, 3 bases, 1 amine, and 1 solvent, at fixed time, temperature, and concentrations. Similarly, the dataset from Perera et al. of 5760 Suzuki reactions^8^ was defined by combinations of 5 electrophiles, 6 nucleophiles, 11 ligands, 7 bases, and 4 solvents. Even a few choices for each component can quickly represent a large experimental space, for which there tends to be a higher cost associated with the HTE campaigns and, particularly with significant numbers of distinct products, a higher analytical burden.

The variation of individual components or aspects of reaction conditions is directly tied to the applicability domain, as a model should not be expected to generalize to a new molecule or input that is too dissimilar from what it has been trained on. As an extreme example, a model that has only seen reactions performed at room temperature cannot understand the influence of temperature on the reaction outcome. In the Ahneman et al. study,^7^ the component with the greatest variation in the dataset was the additive species with 23 total choices, which justifies the evaluation of model generalization to unseen additives in the original paper. With only three bases explored, it is unrealistic to expect the model to anticipate the performance of a fourth unseen base. At the same time, a model trained on a narrow subset of reaction space cannot, in general, be expected to generalize well to other areas of that space, making it vital to select an appropriate set of representative examples.

Mathematically, extrapolation is generally thought of as data that falls outside of the convex hull of training data; however, high-dimensional datasets almost always represent extrapolative tasks by this definition.^12^ The notion of similarity between training and testing points and what constitutes extrapolation in a chemical sense has no strict definition, but distance in chemical feature space (e.g., using descriptors or molecular/reaction fingerprints) is a natural approach. Structural similarity has been used to estimate the domain of applicability and uncertainty of predictive models.^13,14^

## Selecting a Reaction Performance Metric as an Output Variable

There are many commonly reported reaction performance metrics that can be used as the prediction target (output variable) in data-driven models. The two most common are yield, bounded between 0 and 100, and selectivity (e.g., the enantiomeric ratio, regioselectivity, etc.), which is a continuous scalar metric. Other metrics such as the reaction rate or rate constants are less common^15,16^ but are of high interest to process chemists in particular. Rate is a time- and resource-intensive measurement to collect, requiring yields/conversions at many time points. However, rate can be reliably assayed across orders of magnitude and provides insight for practical experimental considerations, such as the reaction concentration, temperature, and time. Enantioselectivity, as reflected by *ΔΔG*^‡^, is a compelling choice for an output variable and has been used in a significant number of successful workflows:^3,17,18^ it is a scalar metric that is centered at 0 when unselective and, due to the relative precision of measuring the enantiomeric ratio (e.r.), does not tend to have a long-tailed distribution. Furthermore, the e.r. most often corresponds to the difference between enantio-determining transition states with the general reaction mechanism otherwise being the same, allowing one to neglect factors that confound modeling yield as an output, such as side reactions or differences in turnover rates of a catalyst. Likewise, regioselectivity is an internally consistent metric that relies only on direct comparisons between candidate atom sites.^19−22^

While selectivity is a useful metric for a subset of reactions, the more universal and widely reported metric in synthetic organic chemistry is yield. Generally, yield prediction has only been successful within large, high-throughput datasets in single/narrow reaction classes. Similar attempts to model diverse literature or “real-world” electronic laboratory notebook (ELN) data produce poorer results given the abundance of confounding variables (e.g., concentrations, time, scale, experimental hardware, the experimentalist) that may be unaccounted for in the reaction description.^23,24^ Different data sources tend to exhibit different distributions of reported reaction yields (Figure 2B).

**Figure 3:**
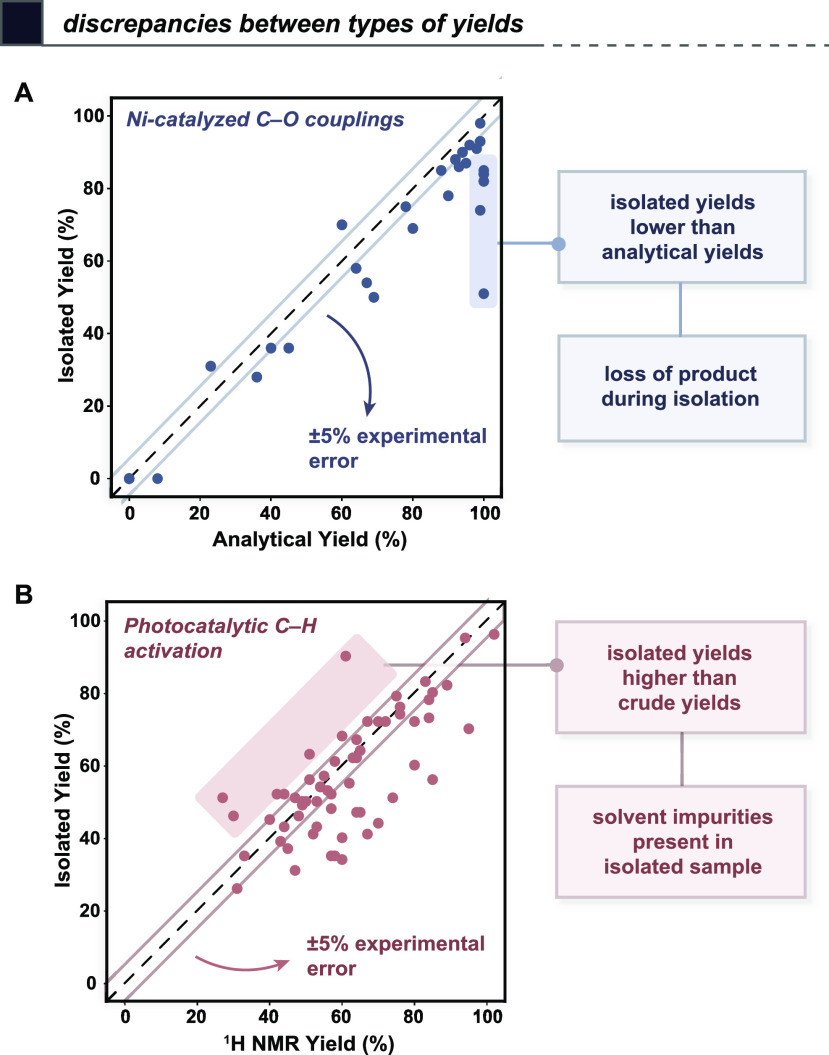
Isolated versus analytical yields for (A) literature-extracted Ni-catalyzed C–O couplings^25^ and (B) a reported photocatalytic C–H activation substrate scope.^26^ Common reasons for the discrepancies between the yields are given.

Reported yields can also be of several types, such as isolated yields, assay yields, or even LCAPs (liquid chromatography area percents), further increasing modeling complexity (Figure 3). Selectivity can suffer from the same ambiguity, but it is more consistently assayed without isolation. LCAP is a common output from HTE campaigns, where it is unrealistic to calibrate yields using product standards for every example. The well-defined range of yield values (0–100%) additionally presents a modeling complication, as many architectures from linear regression to neural networks are able to make predictions outside of this physical range. Compressing or truncating predictions using techniques such as logistic regression or sigmoid activation functions does not tend to improve modeling in our experience. Simplifying the task to a binary (0% versus >0% yield) or categorical (binned yield intervals) classification rather than a regression lowers the analytical burden for data acquisition and mitigates the impact of noise, but it still does not guarantee the ability to train a useful model.

The range of reaction output values represented in a particular dataset will influence the range of output values in its predictions. This is a consideration that is similar to the domain of applicability, where it is necessary to see sufficient diversity during training if one expects it when making predictions. If, for example, the training set has its outputs within a narrow interval (e.g., yields within 70–95%), it is unlikely that the model will be able to make accurate predictions outside of that interval. Common types of models such as random forests (RFs) and Gaussian processes (GPs) are fundamentally incapable of doing so. Multivariate linear models, neural networks, and others can in principle, but their extrapolations will have a higher degree of uncertainty than their interpolations. Nevertheless, studies have shown successful (at times, retrospective) extrapolation during e.r. prediction to select catalysts that achieve selectivity better than anything observed during training.^27−29^ To simplify matters, models that are meant to guide experimental design (e.g., optimize reaction conditions^30^) need not make accurate predictions extrapolating to output values beyond the training set in order to be useful, as evidenced by the success of GPs for Bayesian optimization in chemistry^31^ and beyond.

## Identifying a Molecular/Reaction Representation to Help Define “Diversity”

Supervised learning of complex input/output relationships is the basis of most modeling for chemical reactivity; thus, this goal should guide dataset design. A model’s ability to generalize depends heavily on the representation we use; for example, a categorical (one-hot) encoding of bases does not allow a model to predict the performance of unseen bases, but a representation based on the p*K*_a_ values of the conjugate acids potentially could. If we intend to train a model to understand the impact of base strength, we might plan to run experiments using diverse bases, wherein diversity is defined in terms of base strength, as reflected by the p*K*_a_ of the conjugate acid.

If we hypothesize that there are certain molecular features relevant for modeling, those features should form the basis for defining a diverse set of experiments. This may include using density functional theory (DFT)-based descriptors, which directly capture the electronic and structural properties of molecules that often greatly influence reactivity, or simple physicochemical features such as Mordred descriptors.^32,33^ While the latter type of descriptor is readily calculable with cheminformatics packages in milliseconds, the computational cost of deriving descriptors from DFT calculations can be significant and render these workflows inaccessible or impractical for many researchers. Efforts like kraken^34^ and OSCAR^35^ seek to precompute and/or predict descriptor values for hundreds, thousands, or even millions of hypothetical ligands or catalysts.

A hypothesis-driven approach provides the ability to take an active role in descriptor selection; for example, using a steric parameter and an electronic parameter to define a two-dimensional (2D) array of diverse ligands.^36^ The expert selection of features in this manner introduces bias, which is sometimes beneficial and sometimes detrimental, into dataset generation and modeling. Even when we do not know the importance of different descriptors in a modeling task, however, we can still define diversity with respect to a generic “holistic” set of descriptors. In both settings, the selection of diverse reaction components on the basis of descriptor diversity (through clustering) has been shown to be more informative than those selected less systematically.^16,37^

**Figure 4:**
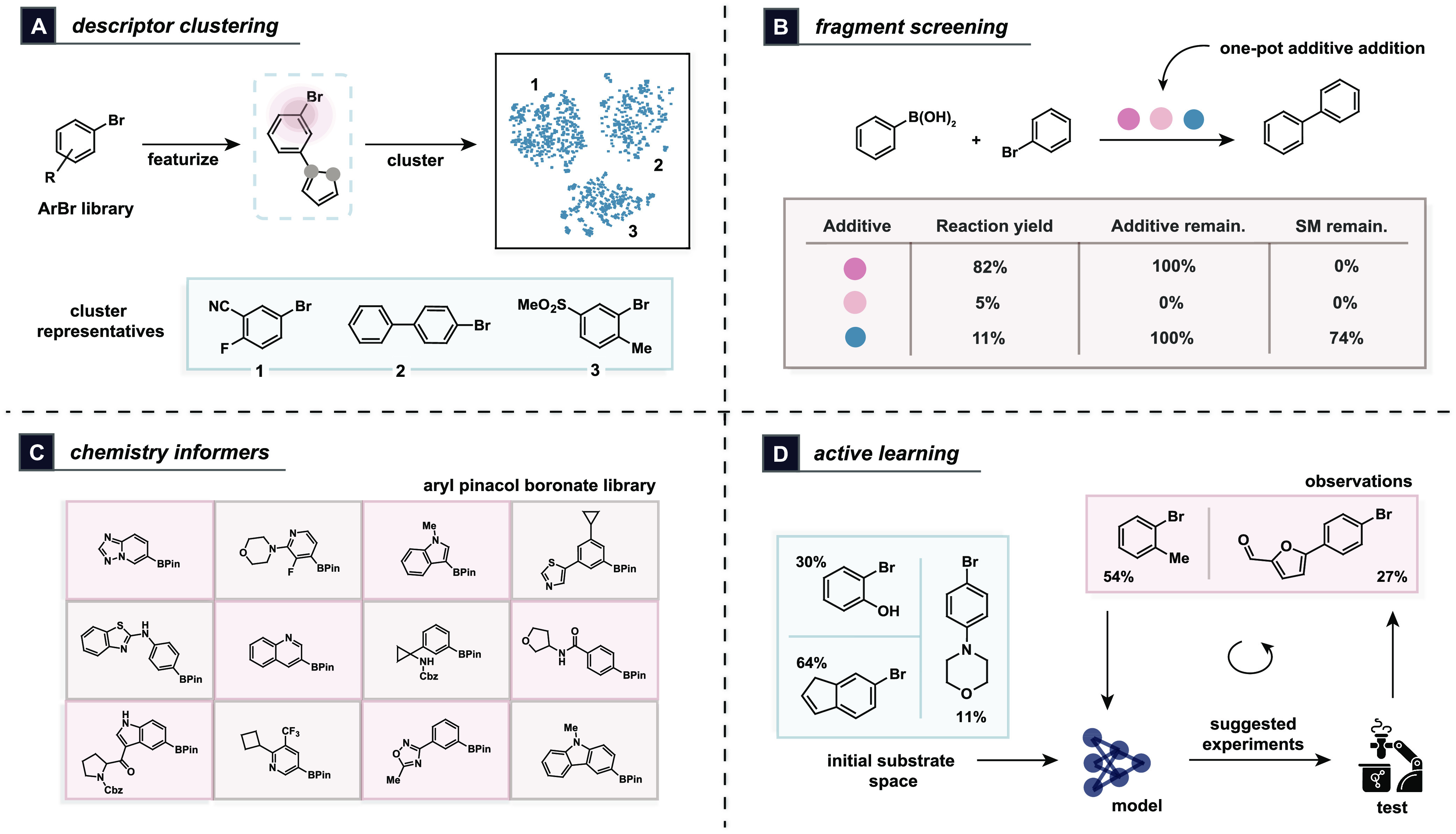
Existing strategies for substrate selection using principles of intentional dataset design. (A) Descriptor-based definition of diverse compound subsets using clustering;^37^ (B) fragment screening to study robustness to additives;^5^ (C) hand-picked structurally diverse chemistry informers;^6^ and (D) active learning to iteratively select experiments based on model predictions.^43^

If we have even less of a prior notion about what will influence reactivity, we can focus on the more abstract “structural diversity”. This might be the case when working with novel reaction types or ones with ambiguous mechanisms. If we anticipate that functional group presence/absence will be most predictive of performance/behavior, we might plan to use a MACCS key,^38^ an extended functional group (EFG),^39^ or another structural fingerprint as the molecular representation in our machine learning model. A dataset can then be designed with this in mind so that experiments directly probe the influence of functional groups on performance. This may be achieved simply by clustering a large set of possible substrates and selecting cluster representatives by using any structural fingerprint representation of choice (Figure 4A). An alternative experimental approach to probing the influence of functional group presence is Glorius robustness screening^5^ (Figure 4B), which uses one-pot addition of several additives to estimate functional group tolerance and preservation in a pooled experiment. Similar high-throughput screenings of additive tolerance have led to an improved understanding of reaction robustness.^40^

The prototypical example of using structural diversity as a proxy for functional diversity (or “synthetic diversity”) is the use of chemistry informer libraries^6,41^ (Figure 4C), as proposed by Merck. One of the original informer libraries is a set of 18 structurally diverse and moderately complex aryl halides meant to sample aryl halide substrates used in medicinal chemistry campaigns. Evaluating substrates with this level of structural complexity might come at the expense of cost, convenience, or interpretability, particularly if an in-house synthesis is required.

Even with the approaches for dataset design we have outlined thus far, identifying useful features might only be possible with a more intimate knowledge of the reaction, particularly for novel chemistry. In such cases, while clustering approaches are still valuable, the selection of substrates that cover the desired domain of applicability can also be done, in principle, in a model-guided manner using active learning.^42^ Rather than selecting the full set of experiments up front, we can use iterative experimental design to train an initial model on a small dataset and then select which hypothetical experiments will be the most informative to perform (Figure 4D). This approach is closely related to Bayesian optimization, but rather than defining the value of an experiment in a manner designed to optimize a performance metric (e.g., yield), the value of an experiment is quantified in a manner designed to maximize model accuracy or minimize uncertainty. Though not commonly employed, active learning for reaction screening has already shown success in retrospective evaluations. Eyke et al. demonstrated that models trained on actively selected subsets of HTE screens can outperform those trained on random subsets.^43^ There have also been efforts to combine active learning and transfer learning for small dataset expansion.^44^ In principle, these approaches have the additional advantage of being able to incorporate existing data, such as literature data, into the model prior to initialization.

## Reflecting on Our Experience: Factors Influencing Success in Reactivity Modeling

### Confounding Variables Lead to Unexplainable Variation in Outcomes

Many attempted prediction tasks are ill-posed. We cannot expect to predict yield values from tabulated literature data when those data do not specify concentrations, purification details, or other essential aspects of reaction conditions; when these are not held constant, they might explain the variance in the output that our models cannot account for. Aggregating multiple existing datasets into a training set for model building is appealing but unlikely to be successful with good accuracy.^45^ This is also true for reaction product prediction, which we and others have, nevertheless, worked on extensively. Using literature data for pretraining and fine-tuning with a designed dataset is also attractive but has not been established as a successful workflow.

### Ambiguous or Noisy Output Variables Obfuscate Reactivity Trends

The meaning of “yield” within one dataset might not match another: does it mean isolated yield, assay yield, LCAP, or conversion? Does 0% mean that no product was formed, or could it mean that it was not able to be isolated or quantified?^46^ Human error, variations in reaction conditions, time dependency, and the effects of purification clashing with the reactivity all complicate modeling efforts. Even within a single dataset, small variations in the reaction and purification conditions may go unreported, further serving to confuse models. Models for the prediction of *ΔΔG*^‡^ or regioselectivity can benefit from error cancellation by focusing on head-to-head comparisons between outcomes.^47^

### Spurious Correlations from Dataset Biases Can Distract Models from the Underlying Chemistry

Confounding variables relating to purification conditions and different data sources, which are particularly present in literature data, hinder models from learning reactivity. For example, recent yield prediction work using a literature-extracted dataset of ∼2000 nickel-catalyzed C–O couplings found that the most important model features implicitly encoded the reaction scale or publication type.^25^ None of these identified features held chemical importance, and the model’s fixation on these extraneous properties likely explains its inability to extrapolate to other substrates.

### Dataset Size Is Often Conflated for Diversity or Coverage

Recent discussions have asserted that a larger substrate scope table is not necessarily more informative than a smaller one.^48^ This is consistent with our experience, where the size of a dataset is not a useful predictor of surrogate model performance. As an example, a larger training set of 37 aryl bromides derived from the literature failed to outperform a dataset of 15 selected via dimensionality reduction and clustering of DFT descriptors in a yield prediction task.^37^

### Combinatorial Design Spaces Are Often Sparse

Yield prediction models trained and evaluated using random splits of combinatorial HTE data work very well. While the initial impression might be that this works because the datasets are large, we argue that it works because it is a simple interpolation (which is clear based on the comparable performance of one-hot representations). Generalization to new species, which is only reflected by certain structured data splits,^49^ is where model limitations are revealed.

The Buchwald–Hartwig^7^ and Suzuki^8^ HTE datasets represent exhaustive explorations of two combinatorial spaces of ∼10^3^ reactions. In contrast, AstraZeneca’s Suzuki ELN dataset^23^ consists of 781 measured reactions out of ∼10^8^ possible combinations of substrates and discrete conditions. The most sparse datasets are, of course, literature-derived datasets, where the millions of known compounds could produce an immeasurably large enumerated space, representing the difficult interpolative and extrapolative challenges for models. While low dataset sparsity seemingly helps models interpolate effectively, it does not ensure extrapolation ability.

### Learning Interactions Is Difficult Even with “Dense” Data

A primary reason sparsity complicates modeling is that interactions are, in general, not additive (though many LFERs add contributions from each component and not their interactions). If the design space of interest is a dense 2D matrix of pairwise substrate combinations, there are techniques that focus on screening small numbers of rows/columns and trying to interpolate,^50^ which is a strategy also used for the validation of building blocks when building DNA-encoded libraries.^51^ However, even models designed to learn interactions may not outperform models that focus on the contributions of each component alone.^52^

### Desiderata Are Not Universal and Depend on Modeling Goals

Modeling reactivity should be done with particular objectives in mind. For example, identifying important descriptors to improve fundamental understanding (interpretability), generalizing small numbers of experiments to combinatorial design spaces (interpolation), predicting the performance of yet untested substrates or catalysts (extrapolation), or guiding experiments in a direction that leads to improved selectivity, yield, etc. (optimization). For each objective, certain simplifying assumptions might be appropriate. Anticipating the performance of novel substrates in a discovery chemistry setting might be compatible with a binary formulation using discretized yields rather than regression. Guiding a yield optimization campaign does not actually require that a model be accurate but merely that the conditions it identifies are promising to lead to improved outcomes. Developing a model without a focused application in mind leads to ambiguity in the importance of different evaluation metrics.

### There Is a Tension in Dataset Design between What Is Ideal for Machine Learning and What Is Tractable to Acquire

## Outlook and Final Practical Recommendations for Dataset Design

The intended representation of a reaction directly informs what a diverse set of input features might look like. In the absence of any prior knowledge, structural or functional diversity (as approximated by descriptors) is defensible; however, as more is known about a particular reaction, focusing on the most relevant features provides an opportunity to maximize the information gained per experiment, e.g., through active learning. Although there is no singular definition of diversity, this paper provides several considerations and recommendations for the field. We summarize our main observations below:

- Formulating problems in terms of selectivity (or relative rate) prediction rather than yield is beneficial; if using yield as the modeling target, avoiding isolated yields can help disentangle reactivity from purification trends.
- Measuring multiple time points provides better opportunities to understand reaction efficiency than measuring single-point yields. A few pilot experiments can help determine the ideal time point(s) at which to measure performance in lieu of a full kinetic profile.
- In the absence of prior knowledge, the selection of the desired reaction component(s) should be done by clustering each component from the design space of interest using computational descriptors or fingerprint representations.
- Generalizable models require more extensive experimental work, both to have sufficient data to train and to perform structured extrapolative evaluations.
- Active learning is the most principled approach for selecting substrates or conditions to evaluate if experimental capacity is limited. Relevant literature data, while not the main focus of this Outlook, can be used at the outset of an active learning campaign.

The ideal “dataset design” will always be a moving target that depends on our modeling goals and experimental capabilities. We encourage readers to explore and adopt more systematic ways of designing HTE campaigns and substrate scope tables.

## References

[ref1] Hammett L. P. The Effect of Structure upon the Reactions of Organic Compounds. Benzene Derivatives. J. Am. Chem. Soc. 1937, 59 (1), 96–103. 10.1021/ja01280a022.

[ref2] Williams W. L.; Zeng L.; Gensch T.; Sigman M. S.; Doyle A. G.; Anslyn E. V. The Evolution of Data-Driven Modeling in Organic Chemistry. ACS Cent. Sci. 2021, 7 (10), 1622–1637. 10.1021/acscentsci.1c00535. 34729406 PMC8554870

[ref3] Sigman M. S.; Harper K. C.; Bess E. N.; Milo A. The Development of Multidimensional Analysis Tools for Asymmetric Catalysis and Beyond. Acc. Chem. Res. 2016, 49 (6), 1292–1301. 10.1021/acs.accounts.6b00194. 27220055

[ref4] Tu Z.; Stuyver T.; Coley C. W. Predictive Chemistry: Machine Learning for Reaction Deployment, Reaction Development, and Reaction Discovery. Chem. Sci. 2023, 14 (2), 226–244. 10.1039/D2SC05089G. 36743887 PMC9811563

[ref5] Collins K. D.; Glorius F. A Robustness Screen For The Rapid Assessment of Chemical Reactions. Nat. Chem. 2013, 5 (7), 597–601. 10.1038/nchem.1669. 23787750

[ref6] Kutchukian P. S.; Dropinski J. F.; Dykstra K. D.; Li B.; DiRocco D. A.; Streckfuss E. C.; Campeau L.-C.; Cernak T.; Vachal P.; Davies I. W.; et al. Chemistry Informer Libraries: a Chemoinformatics Enabled Approach to Evaluate and Advance Synthetic Methods. Chem. Sci. 2016, 7 (4), 2604–2613. 10.1039/C5SC04751J. 28660032 PMC5477042

[ref7] Ahneman D. T.; Estrada J. G.; Lin S.; Dreher S. D.; Doyle A. G. Predicting Reaction Performance in C–N Cross-Coupling Using Machine Learning. Science 2018, 360 (6385), 186–190. 10.1126/science.aar5169. 29449509

[ref8] Perera D.; Tucker J. W.; Brahmbhatt S.; Helal C. J.; Chong A.; Farrell W.; Richardson P.; Sach N. W. A Platform for Automated Nanomole-Scale Reaction Screening and Micromole-Scale Synthesis in Flow. Science 2018, 359 (6374), 429–434. 10.1126/science.aap9112. 29371464

[ref9] King-Smith E.; Berritt S.; Bernier L.; Hou X.; Klug-McLeod J.; Mustakis J.; Sach N.; Tucker J.; Yang Q.; Howard R.; et al. Probing the Chemical “Reactome” with High Throughput Experimentation Data. ChemRxiv 2023, 1 10.26434/chemrxiv-2022-hjnmr. PMC10997498 38168924

[ref10] Mahjour B.; Hoffstadt J.; Cernak T. Designing Chemical Reaction Arrays Using Phactor and ChatGPT. Org. Process Res. Dev. 2023, 27 (8), 1510–1516. 10.1021/acs.oprd.3c00186.

[ref11] Reich M.; Schunk S.; Jostock R.; De Vry J.; Kneip C.; Germann T.; Engels M. Preparation of substituted benzamide compounds for treating conditions mediated at least in part via the bradykinin 1 receptor. U.S. Patent 20120071461, 2012.

[ref12] Balestriero R.; Pesenti J.; LeCun Y. Learning in High Dimension Always Amounts to Extrapolation. arXiv 2021, 1 10.48550/arXiv.2110.09485.

[ref13] Toplak M.; Močnik R.; Polajnar M.; Bosnić Z.; Carlsson L.; Hasselgren C.; Demšar J.; Boyer S.; Zupan B.; Stålring J. Assessment of Machine Learning Reliability Methods for Quantifying the Applicability Domain of QSAR Regression Models. J. Chem. Inf. Model. 2014, 54 (2), 431–441. 10.1021/ci4006595. 24490838

[ref14] Rakhimbekova A.; Madzhidov T. I.; Nugmanov R. I.; Gimadiev T. R.; Baskin I. I.; Varnek A. Comprehensive Analysis of Applicability Domains of QSPR Models for Chemical Reactions. Int. J. Mol. Sci. 2020, 21 (15), 5542 10.3390/ijms21155542. 32756326 PMC7432167

[ref15] Lu J.; Paci I.; Leitch D. C. A Broadly Applicable Quantitative Relative Reactivity Model for Nucleophilic Aromatic Substitution (SNAr) Using Simple Descriptors. Chem. Sci. 2022, 13 (43), 12681–12695. 10.1039/D2SC04041G. 36519044 PMC9645419

[ref16] Haas B. C.; Goetz A. E.; Bahamonde A.; McWilliams J. C.; Sigman M. S. Predicting Relative Efficiency of Amide Bond Formation Using Multivariate Linear Regression. Proc. Natl. Acad. Sci. U.S.A. 2022, 119 (16), e2118451119 10.1073/pnas.2118451119. 35412905 PMC9169781

[ref17] Crawford J. M.; Kingston C.; Toste F. D.; Sigman M. S. Data Science Meets Physical Organic Chemistry. Acc. Chem. Res. 2021, 54 (16), 3136–3148. 10.1021/acs.accounts.1c00285. PMC9078128 34351757

[ref18] Reid J. P.; Sigman M. S. Holistic Prediction of Enantioselectivity in Asymmetric Catalysis. Nature 2019, 571 (7765), 343–348. 10.1038/s41586-019-1384-z. 31316193 PMC6641578

[ref19] Guan Y.; Coley C. W.; Wu H.; Ranasinghe D.; Heid E.; Struble T. J.; Pattanaik L.; Green W. H.; Jensen K. F. Regio-selectivity Prediction with a Machine-Learned Reaction Representation and n-the-fly Quantum Mechanical Descriptors. Chem. Sci. 2021, 12 (6), 2198–2208. 10.1039/D0SC04823B. PMC8179287 34163985

[ref20] Guan Y.; Lee T.; Wang K.; Yu S.; McWilliams J. C. SNAr Regioselectivity Predictions: Machine Learning Triggering DFT Reaction Modeling through Statistical Threshold. J. Chem. Inf. Model. 2023, 63 (12), 3751–3760. 10.1021/acs.jcim.3c00580. 37272922

[ref21] Nippa D. F.; Atz K.; Hohler R.; Müller A. T.; Marx A.; Bartelmus C.; Wuitschik G.; Marzuoli I.; Jost V.; Wolfard J.; et al. Enabling Late-Stage Drug Diversification by High-Throughput Experimentation with Geometric Deep Learning. ChemRxiv 2022, 1 10.26434/chemrxiv-2022-gkxm6. PMC10849962 37996732

[ref22] Caldeweyher E.; Elkin M.; Gheibi G.; Johansson M.; Sköld C.; Norrby P.-O.; Hartwig J. F. Hybrid Machine Learning Approach to Predict the Site Selectivity of Iridium-Catalyzed Arene Borylation. J. Am. Chem. Soc. 2023, 145 (31), 17367–17376. 10.1021/jacs.3c04986. 37523755 PMC11723321

[ref23] Saebi M.; Nan B.; Herr J. E.; Wahlers J.; Guo Z.; Zuranski A. M.; Kogej T.; Norrby P.-O.; Doyle A. G.; Chawla N. V.; Wiest O. On the Use of Real-World Datasets for Reaction Yield Prediction. Chem. Sci. 2023, 14 (19), 4997–5005. 10.1039/D2SC06041H. 37206399 PMC10189898

[ref24] Schwaller P.; Vaucher A. C.; Laino T.; Reymond J.-L. Prediction of Chemical Reaction Yields Using Deep Learning. Mach. Learn.: Sci. Technol. 2021, 2 (1), 015016 10.1088/2632-2153/abc81d.

[ref25] Schleinitz J.; Langevin M.; Smail Y.; Wehnert B.; Grimaud L.; Vuilleumier R. Machine Learning Yield Prediction from NiCOlit, a Small-Size Literature Data Set of Nickel Catalyzed C–O Couplings. J. Am. Chem. Soc. 2022, 144 (32), 14722–14730. 10.1021/jacs.2c05302. 35939717

[ref26] Ruos M. E.; Kinney R. G.; Ring O. T.; Doyle A. G. A General Photocatalytic Strategy for Nucleophilic Amination of Primary and Secondary Benzylic C–H Bonds. J. Am. Chem. Soc. 2023, 145 (33), 18487–18496. 10.1021/jacs.3c04912. 37565772

[ref27] Zahrt A. F.; Henle J. J.; Rose B. T.; Wang Y.; Darrow W. T.; Denmark S. E. Prediction of Higher-Selectivity Catalysts by Computer-Driven Workflow and Machine Learning. Science 2019, 363 (6424), eaau5631 10.1126/science.aau5631. 30655414 PMC6417887

[ref28] Liles J. P.; Rouget-Virbel C.; Wahlman J. L. H.; Rahimoff R.; Crawford J. M.; Medlin A.; O’Connor V. S.; Li J.; Roytman V. A.; Toste F. D.; et al. Data Science Enables the Development of a New Class of Chiral Phosphoric Acid Catalysts. Chem. 2023, 9 (6), 1518–1537. 10.1016/j.chempr.2023.02.020. 37519827 PMC10373836

[ref29] Zhao S.; Gensch T.; Murray B.; Niemeyer Z. L.; Sigman M. S.; Biscoe M. R. Enantiodivergent Pd-Catalyzed C–C Bond Formation Enabled through Ligand Parameterization. Science 2018, 362 (6415), 670–674. 10.1126/science.aat2299. 30237245 PMC6613548

[ref30] Reizman B. J.; Jensen K. F. Feedback in Flow for Accelerated Reaction Development. Acc. Chem. Res. 2016, 49 (9), 1786–1796. 10.1021/acs.accounts.6b00261. 27525813

[ref31] Shields B. J.; Stevens J.; Li J.; Parasram M.; Damani F.; Alvarado J. I. M.; Janey J. M.; Adams R. P.; Doyle A. G. Bayesian Reaction Optimization as a Tool for Chemical Synthesis. Nature 2021, 590 (7844), 89–96. 10.1038/s41586-021-03213-y. 33536653

[ref32] Milo A.; Bess E. N.; Sigman M. S. Interrogating Selectivity in catalysis using Molecular Vibrations. Nature 2014, 507 (7491), 210–214. 10.1038/nature13019. 24622199

[ref33] Gallegos L. C.; Luchini G.; St John P. C.; Kim S.; Paton R. S. Importance of Engineered and Learned Molecular Representations in Predicting Organic Reactivity, Selectivity, and Chemical Properties. Acc. Chem. Res. 2021, 54 (4), 827–836. 10.1021/acs.accounts.0c00745. 33534534

[ref34] Gensch T.; Dos Passos Gomes G.; Friederich P.; Peters E.; Gaudin T.; Pollice R.; Jorner K.; Nigam A.; Lindner-D’Addario M.; Sigman M. S.; et al. A Comprehensive Discovery Platform for Organophosphorus Ligands for Catalysis. J. Am. Chem. Soc. 2022, 144 (3), 1205–1217. 10.1021/jacs.1c09718. 35020383

[ref35] Gallarati S.; van Gerwen P.; Laplaza R.; Vela S.; Fabrizio A.; Corminboeuf C. OSCAR: an Extensive Repository of Chemically and Functionally Diverse Organocatalysts. Chem. Sci. 2022, 13 (46), 13782–13794. 10.1039/D2SC04251G. 36544722 PMC9710326

[ref36] Bess E. N.; Bischoff A. J.; Sigman M. S. Designer Substrate Library for Quantitative, Predictive Modeling of Reaction Performance. Proc. Natl. Acad. Sci. U. S. A. 2014, 111 (41), 14698–14703. 10.1073/pnas.1409522111. 25267648 PMC4205662

[ref37] Kariofillis S. K.; Jiang S.; Żurański A. M.; Gandhi S. S.; Martinez Alvarado J. I.; Doyle A. G. Using Data Science To Guide Aryl Bromide Substrate Scope Analysis in a Ni/Photoredox-Catalyzed Cross-Coupling with Acetals as Alcohol-Derived Radical Sources. J. Am. Chem. Soc. 2022, 144 (2), 1045–1055. 10.1021/jacs.1c12203. 34985904 PMC8810294

[ref38] Durant J. L.; Leland B. A.; Henry D. R.; Nourse J. G. Reoptimization of MDL Keys for Use in Drug Discovery. J. Chem. Inf. Comput. Sci. 2002, 42 (6), 1273–1280. 10.1021/ci010132r. 12444722

[ref39] Salmina E. S.; Haider N.; Tetko I. V. Extended Functional Groups (EFG): An Efficient Set for Chemical Characterization and Structure-Activity Relationship Studies of Chemical Compounds. Molecules 2016, 21 (1), 1 10.3390/molecules21010001. PMC6273096 26703557

[ref40] Prieto Kullmer C. N.; Kautzky J. A.; Krska S. W.; Nowak T.; Dreher S. D.; MacMillan D. W. C. Accelerating Reaction Generality and Mechanistic Insight through Additive Mapping. Science 2022, 376 (6592), 532–539. 10.1126/science.abn1885. 35482871 PMC9673503

[ref41] Dreher S. D.; Krska S. W. Chemistry Informer Libraries: Conception, Early Experience, and Role in the Future of Cheminformatics. Acc. Chem. Res. 2021, 54 (7), 1586–1596. 10.1021/acs.accounts.0c00760. 33723992

[ref42] Settles B. In Active Learning Literature Survey. 2009. https://burrsettles.com/pub/settles.activelearning.pdf (accessed 2023-09-01).

[ref43] Eyke N. S.; Green W. H.; Jensen K. F. Iterative Experimental Design Based on Active Machine Learning Reduces the Experimental Burden Associated with Reaction Screening. React. Chem. Eng. 2020, 5 (10), 1963–1972. 10.1039/D0RE00232A.

[ref44] Shim E.; Tewari A.; Cernak T.; Zimmerman P. M. Machine Learning Strategies for Reaction Development: Toward the Low-Data Limit. J. Chem. Inf. Model. 2023, 63 (12), 3659–3668. 10.1021/acs.jcim.3c00577. 37312524 PMC11163943

[ref45] Strieth-Kalthoff F.; Sandfort F.; Kühnemund M.; Schäfer F. R.; Kuchen H.; Glorius F. Machine Learning for Chemical Reactivity: The Importance of Failed Experiments. Angew. Chem., Int. Ed. 2022, 61 (29), e202204647 10.1002/anie.202204647. 35512117

[ref46] Maloney M. P.; Coley C. W.; Genheden S.; Carson N.; Helquist P.; Norrby P.-O.; Wiest O. Negative Data in Data Sets for Machine Learning Training. J. Org. Chem. 2023, 88 (9), 5239–5241. 10.1021/acs.joc.3c00844. 37126471

[ref47] Xu J.; Grosslight S.; Mack K. A.; Nguyen S. C.; Clagg K.; Lim N.-K.; Timmerman J. C.; Shen J.; White N. A.; Sirois L. E.; et al. Atroposelective Negishi Coupling Optimization Guided by Multivariate Linear Regression Analysis: Asymmetric Synthesis of KRAS G12C Covalent Inhibitor GDC-6036. J. Am. Chem. Soc. 2022, 144 (45), 20955–20963. 10.1021/jacs.2c09917. 36326518

[ref48] Kozlowski M. C. On the Topic of Substrate Scope. Org. Lett. 2022, 24 (40), 7247–7249. 10.1021/acs.orglett.2c03246. 36239031

[ref49] Zahrt A. F.; Henle J. J.; Denmark S. E. Cautionary Guidelines for Machine Learning Studies with Combinatorial Datasets. ACS Comb. Sci. 2020, 22 (11), 586–591. 10.1021/acscombsci.0c00118. 33000621

[ref50] Xu J.; Kalyani D.; Struble T.; Dreher S.; Krska S.; Buchwald S. L.; Jensen K. F. Roadmap to Pharmaceutically Relevant Reactivity Models Leveraging High-Throughput Experimentation. ChemRxiv 2022, 1 10.26434/chemrxiv-2022-x694w. PMC13047508 40439243

[ref51] Hudson L.; Mason J. W.; Westphal M. V.; Richter M. J. R.; Thielman J. R.; Hua B. K.; Gerry C. J.; Xia G.; Osswald H. L.; Knapp J. M.; et al. Diversity-Oriented Synthesis Encoded by Deoxyoligonucleotides. Nat. Commun. 2023, 14 (1), 4930 10.1038/s41467-023-40575-5. 37582753 PMC10427684

[ref52] Goldman S.; Das R.; Yang K. K.; Coley C. W. Machine Learning Modeling of Family Wide Enzyme-Substrate Specificity Screens. PLoS Comput. Biol. 2022, 18 (2), e1009853 10.1371/journal.pcbi.1009853. 35143485 PMC8865696

